# The Use of Balanced Scorecards in Mental Health Services: an Integrative Review and Thematic Analysis

**DOI:** 10.1007/s11414-022-09806-3

**Published:** 2022-07-14

**Authors:** Rachel E. Brimelow, Aneline Amalathas, Elizabeth Beattie, Gerard Byrne, Nadeeka N. Dissanayaka

**Affiliations:** 1 The University of Queensland Centre for Clinical Research, Herston, QLD 4029 Australia; 2 The University of Queensland-Ochsner Clinical School, Brisbane, Australia; 3 Ochsner Clinical School, The University of Queensland, New Orleans, LA USA; 4 Faculty of Health, School of Nursing, Queensland University of Technology, Kelvin Grove, QLD 4059 Australia; 5 Institute of Health and Biomedical Innovation, Kelvin Grove, QLD 4059 Australia; 6 Mental Health Service, Royal Brisbane & Woman’s Hospital, Herston, QLD 4029 Australia; 7 Faculty of Health and Behavioural Sciences, The University of Queensland School of Psychology, St Lucia, QLD 4072 Australia; 8 Department of Neurology, Royal Brisbane and Women’s Hospital, Herston, QLD 4029 Australia

**Keywords:** Balanced scorecard, Governance, Mental health, Quality of care

## Abstract

Performance management of mental health services (MHS) through quality reporting of strategic indicators and goals is essential to improve efficiency and quality of care. One such method is the balanced scorecard (BSC). This integrative review of peer-reviewed and industry implemented BSCs in MHS aims to inform future development of a more comprehensive mental health–focused benchmarking tool. A two-part systematic literature search consisted of peer-reviewed published literature on MHS specific BSCs utilising the PRISMA guidelines in addition to industry published BSCs available online. A total of 17 unique BSCs were identified. A total of 434 indicators were subject to thematic analysis identifying 11 key themes: prevalence, accessibility, services provided, clinical outcomes, client satisfaction, client involvement, staff motivation, staffing levels, governance and compliance, development, and costs and revenue. These themes represented the measures that MHS believed measured key performance criteria in alignment with their organisational objectives.

## Introduction

The prevalence of mental health disorders across the globe represents a significant challenge to health care services. Lifetime prevalence estimates range from 32 to 85% with longitudinal studies revealing that most people will suffer a diagnosable mental disorder at some stage during their lifetime.^1^ The high prevalence of mental health conditions has led to significant health expenditure and health service utilisation.^2,3^ Consequently, measures of mental health care service provision and quality of care have been sought. Performance evaluation in health care services is difficult due to the dynamic nature of the industry, with complexities surrounding patient perceived quality of care, responsiveness, and social responsibilities, leading to increased costs and inefficiencies.^4,5^ Numerous interlinking personnel and services involved in MHS contributes to system complexity and patient flow.^6^

Evidence-based measures utilised for performance management has lagged within the MHS sector.^7^ Whilst performance measurement systems required for accreditation and quality improvement generally include measures of structure, process, and outcomes, it is postulated that a balanced portfolio of measures across these categories is required.^7^ Health services require availability of resources, internal procedures, and sufficient workforce to deliver and evaluate evidence-based practice.^8^ Although performance management within MHS has improved with the development of the World Health Organization (WHO)’s Assessment Instrument for Mental Health Systems targeted at the broader system level of high income countries,^9^ performance measurement at the individual MHS level still relies on organisational strategy. One measure of system performance that has been deployed in the health care context at this MHS level is the balanced scorecard (BSC).

The BSC tracks measures of performance that reflect the strategic objectives of an organisation and can be considered as a tool for performance measurement, strategy evaluation, and communication.^10^ It moves away from traditional fiscal measures of organisational success to include measures of intangible assets and intellectual capital that more accurately reflect the scope of the organisation.^11^ Originally developed by Kaplan and Norton, the BSC tracks metrics and indicators that align with the organisation’s strategic objectives across four perspectives—financial, customer, internal, and learning and growth.^12,13^ Organisational management can interpret this data to measure short- and long-term performance in the specified domains and develop strategies to increase improvement and growth.^14^

The BSC has evolved over time with three major iterations or ‘generations’ described in the literature:^15,16^ (1) First-generation BSCs prioritised multidimensional measurement of business performance across the four perspectives with a focus on stakeholder satisfaction; (2) second-generation BSCs included the notion that causal relationships between objectives could be identified with the introduction of strategic maps;^16^ and (3) third-generation BSCs moved towards a strategic linkage model focused on outcomes and activities moving away from the traditional four perspectives.^17^ Literature reporting the implementation and sources of BSCs in industry most often refer to the first two iterations.^15^

## Theory

Health care institutions utilise the BSC to measure organisational performance and efficacy of care provided, particularly with the advent of integrated health care centres that have numerous goals, stakeholders, and strategies for success.^14^ Studies on the implementation of BSCs in the health care context have shown they can successfully provide performance benchmarking of health service capacity and service delivery, as well as stimulating new dialogue about organisational vision and strategy to instigate change.^18,19^ A review on the utilisation of BSCs in health care has highlighted their usefulness in the individual organisational context including aiding transition during organisational change and providing a basis for quality improvement/staff competence initiatives and as a framework to improve clinical governance.^20^ This underlines the importance of the BSC as a communication and strategy evaluation tool in addition to performance management.

Case studies have described how information generated from the BSC can influence alignment of clinical care to organisational goals. For example, the implementation of a BSC in an American teaching hospital led to greater awareness by clinicians of their roles in the success of the hospital’s organisational strategies and goals (beyond their immediate roles in clinical care), and highlighted the interconnectedness of those institutional goals and quality of care outcomes.^21^ Whilst the majority of BSC implementation case studies have been in academic centres, there are examples of health care system-wide usage including the Dutch health system and a United States Department of Veterans Affairs health care system.^22,23^ Striking the appropriate balance between individualised strategic needs and baseline consistency between iterations appears to be key to successful widespread implementation.^24^ The existence of such variety demonstrates enthusiasm for BSC use in health care. However, less in known about their development and implementation in MHS.

This review will examine the use of BSC in mental health care management. Utilisation of the BSC in MHS is a growing trend, though still in its nascence compared to usage in non-mental health institutions. Although a 1999 paper argued for the benefits of applying the BSC to behavioural health care to measure financial and quality-of-care outcomes, the author acknowledged the difficulties in selecting the appropriate indicators at such an early stage of health care information systems.^25^ Since then, efforts have been made toward development and implementation of the BSC in the domain of mental health at numerous institutions. Here, the authors present the results of a systematic review of the development and implementation of the BSC in mental health care with the aim of providing useful information about the key components and informing future development.

## Methodology

Initial searches revealed a paucity of peer-reviewed literature on the implementation of BSCs in the mental health sector; however, industry reports suggested much wider adoption of the tool. A two-part strategy to review the use of BSC in MHS was therefore developed to provide a more comprehensive review. Part A focused on peer-reviewed literature on the development and/or implementation of BSCs in the MHS sector. Part B focused on industry reporting of BSC development or results in MHS. Two authors were independently responsible for inclusion/exclusion of articles in part A and part B, where differences in article inclusion was observed consensus was reached.

### Part A

A literature review of published journal articles was conducted following the PRIMSA guidelines.^26^ Databases searched included PubMed, Embase, and CINAHL. Papers were included if they were a research article published from 2009 to 2019, available in English, peer-reviewed, and directly addressed the development or use of BSCs in MHS or organisations. Iterations of BSCs from one to three were included. Papers were excluded if both strategic objectives and indicators were not reported, or if the scorecard did not meet the criteria for a BSC.

Search terms included:

- Pubmed: Scorecard[tiab] AND “mental health”[MeSH Terms] OR “mental”[All Fields] AND “health”[All Fields] OR “mental health”[All Fields] OR scorecard[tiab] AND psych [All Fields].
- Embase: Scorecard AND mental AND (‘health’/exp OR health) AND (‘care’/exp OR care) OR scorecard NEAR/10 psych*
- CINAHL: TI scorecard OR AB scorecard AND psych* OR mental

**Fig. 1:**
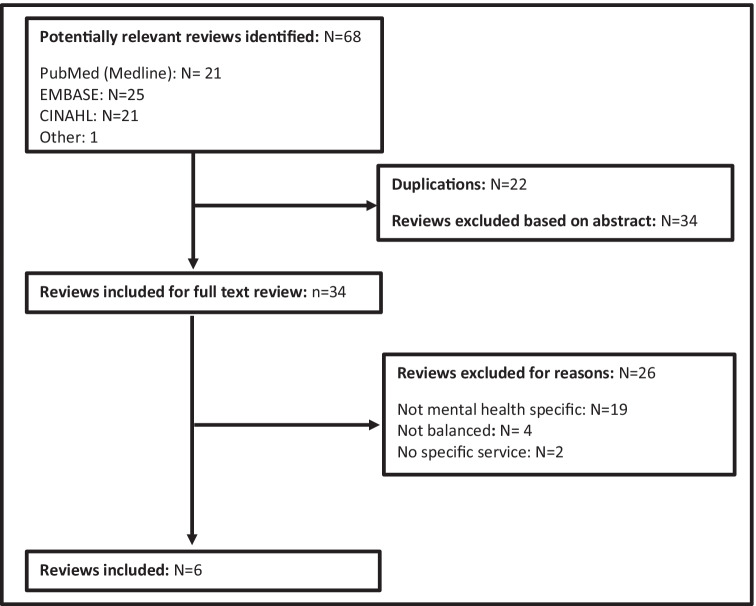
Study screening process for peer-reviewed BSCs

The screening process for inclusion and exclusion of peer-reviewed BSC articles is presented in Fig. 1.

### Part B

Grey literature was searched for current BSCs implemented in MHS around the world. BSCs had to be available in the public forum, online, and accessible in December 2018 to January 2019. Iterations of BSC one to three were included. Search strategy is represented in Table 1.

**Table 1:** Search strategy for industry BSCs (grey literature)

Search	Search terms	Google results generated	Pages accessed	BSC identified
1	Balanced scorecard + mental health	964,000	10	11
2	Scorecard + mental health services	3,300,000	10	3
3	Performance indicators + mental health	41,200,000	10	0
4	Balanced scorecard + psychiatry	360,000	10	3
5	Balanced scorecard + behavioural health	3,200,000	10	1
6	Balanced scorecard + mental health services	2,760,000	10	1
7	Scorecard + mental health	2,740,000	10	0
8	Scorecard + psychiatry	299,000	10	1
9	Scorecard + behavioural health	1,250,000	10	0
10	Performance indicators + psychiatry	23,800,000	10	0
11	Performance indicators + behavioural health	120,000,000	10	0
			**Total**	**20**
			**Excluded**	**9**
			**Included**	**11**

### Data Analysis

Where BSCs published the indicators used to measure level of success of the service in relation to their core objectives, indicators were collated and utilised for thematic analysis. Inductive thematic analysis was used to produce coded categories derived directly from the text data.^27^ Themes were defined as organised groups of repeating ideas within the text that provided meaning at the interpretative level. Examination of the data revealed 11 distinct themes arising from the reported indicators. Each indicator was subsequently coded into one of 12 categories (11 themes and a miscellaneous category). BSCs were then analysed for the presence and frequency of coded indicators to identify the key themes present in each BSC. This was conducted independently by two authors and consensus reached.

Examination for risk of bias using accepted tools was not appropriate for the unique data set; however, the authors accept the possibility of publishing bias in terms of BSCs presented to the public by health services.

## Results

A total of 17 unique BSCs were identified from search strategies A and B. Review of peer-reviewed literature revealed 6 BSCs within the MHS from the USA, Canada, the UK, New Zealand, and Singapore (Table 2). A further 11 unique BSCs were identified in the grey literature (part B) across Ireland, the USA, Canada, and the UK (Table 3). Overall, there were 14 MHS identified as using a BSC approach in the grey literature.

**Table 2:** Summary of balanced scorecards in mental health services within academic literature (part A)

Author	Setting	Strategic goals—summary	Domains	Indicators
**Part A**				
Coop, 2006	Mental Health Service, New Zealand (non-profit-GO)	Not stated	Clinical quality; productivity; learning and organisational; financial	21
Chong 2007, 2008	Early Psychosis Intervention Program, Singapore (non-profit-GO)	Detect and manage psychosis early through screening; improve quality of life of those with psychosis; raise awareness of early signs and symptoms of psychosis; establish strong link with primary health care providers; reduce stigma	Customer; internal process; employee learning and growth; financial	-
Lin & Durbin, 2008	Mental health and Addictions Inpatient Care, Canada (non-profit-GO)	Targeted and appropriate use of inpatient services; comprehensive continuum of services and supports; services based on current best practices; consumer-centred care	Patient perception of care; clinical utilisation; system integration and change; financial performance	29
Schmidt et al., 2006	Mental Health Trust, UK (non-profit-GO)	Improve conditions of service users; minimise risk of harm; Provide appropriate service (right time, right place); pay attention employee health; train and inform employees; ensure balanced financial position; control additional workforce costs	Clinical risk; service modernisation; workforce; finance	23
Wolferstieg & Dunham, 1998	Hudson River Psychiatric Centre, USA (non-profit-GO)	Develop product lines requiring re-organisation of inpatient services; develop staff training and competence assessment plan; develop performance measurement; increase staff’s fiscal awareness of costs of activities; automation of medical records; develop proactive relations plan; increase stakeholder awareness of safety and environmental issues; reduce rates of restrictive practices and occupational injuries	Financial; customer; innovation and learning; internal business	15
Yang et al., 2016	Child and Youth Mental health System, Canada (non-profit-GO)	Not stated	Known prevalence; system use; outcomes; access; quality; early identification	25

*GO*, government lead organisation; *NGO*, non-government lead organisation

**Table 3:** Summary of mental health services industry–balanced scorecards within grey literature (part B)

Author	Setting	Strategic goals—summary	Domains	Indicators
NHS Scotland CAMHS, 2011	Child and Adolescent Mental Health, UK (non-profit-GO)	Person centred—patient values guide all clinical decision; safe—providing services based on scientific knowledge; effective—avoiding waste; equitable—providing care that does not vary in quality; timely—reducing waits and sometimes harmful delays	Client/patient focus; delivering best practice; internal process; best use of resources	18
Ontario Shores Centre for Mental Health Sciences, 2019	Mental Health Service, Canada (non-profit-GO)	Develop a coordinated mental health and addictions system to provide greater access; give our people the freedom to be innovative; weave recovery into the fabric of our organisation; set the highest standards for practice, quality and performance	Customer; hospital process; learning and growth; financial	17
CMHA Toronto, 2011	Canadian Mental Health association, Canada (non-profit-GO)	Continue prudent fiscal management; develop and implement new fundraising strategy; continue advocacy and system leadership; promote mental health and understanding; implement diversity and equity plan; develop and embed consumer participation strategies; service high need areas; develop chronic disease prevention and management options; develop concurrent disorder capacity; ensure CMHA remains a great place to work; develop quality and safety improvement; develop a learning culture; accreditation	Finance; client and community; internal processes; learning and growth	28
Western Isles Health and Social Care, 2018	NHS, Western Isles, UK (non-profit-GO)	Develop system allocating personal budgets to social care needs; adult support and protection protocols strengthened; multi-disciplinary teams deliver coordinated care; invest in technology and improve processes; delegate resources to alcohol and drug partnership; develop a 3-year workforce plan; deliver intermediate care service to prevent hospital admission and support discharge; develop intensive rehabilitation service; establish health and social care hub in every locality; implement Scottish Patient Safety Programme; reduce number of long-term placement within off-island health and social care facilities; reduce expenditure; support GPs to have multi-disciplinary teams; support people with long-term conditions to self-manage; deliver integrated community model with recovery focus; support community to tackle social isolation; ensure carers has care support plans	Quality of care—person-centred; quality of care—safe and supported; population health; value and sustainability	94
New Path youth and family services, 2018	New Path Youth and Family Mental Health Services, Canada (non-profit NGO)	Not stated	Program priority; people priority; partnership priority; finance and administration priority	11
Mental Health Ireland; 2017	Mental health Ireland (non-profit-GO)	Maximising the value of our work; developing our network; ensuring our sustainability	Financial and corporate management; service delivery; learning and growth; client and stakeholders	23
Alaska Mental Health Trust Authority, 2019	Mental Health Trust Authority, Alaska USA (non-profit-GO)	Not stated	Health; safety; living with dignity; economic security	23
Centre for Addiction and Mental Health; The Royal Mental Health Centre; Ontario Shores; Waypoint, 2019	Mental Health and Addictions Quality Initiative Peer Scorecard, Canada (non-profit-GO)	Medication safety—increase outpatients receiving medication reconciliation after discharge; workplace violence prevention; reduce use of physical restraints; reduce wait time in the emergence department and EAU; improve patient satisfaction; reduce % hospital readmissions within 7 days of discharge; increase % of patients with collected demographic information	Client complexity; client outcomes; client safety; client access; staff safety; HR indicator; financial	17
Griffin Center, 2018	Griffin Center balanced scorecard, Canada (non-profit NGO)	Partner to improve community care; impact through innovation; optimise our resources; build on our strengths; continuously improve; attract, retain, and develop talent; enhanced client lives; lead in pour community; build a sustainable resource base sustained giving	Stakeholder; financial; internal organisational; learning and growth	21
Spectra helpline Canada, 2017	Spectra helpline services for community and mental health, Peel Canada (non-profit NGO)	Improve access to support and intervention; improve access to multilingual service; diversify service channel; use community engagement strategies to inform the community of programs/services; reduce stigma; pursue shared mission; standardise human resources policies and staff procedures; encourage ambitions of staff; positon spectra as organisation of choice for student placement; have reputation for evidence-based best practice; cultivate climate of continual improvement; enhance ability to capture and measure client and community outcomes; be transparent; determine social ROI; select volunteer with skillset and competencies; increase volunteer quality of training and educational experiences; increase/improve language line volunteer training; improved information management and technology; establish brand and identity; increase total value of funding; explore social enterprise opportunities; embark in partnerships that extend expertise/broaden reach	Help people cope and build resiliency; a great place to work and develop; dedicated to being the best we can be; operational excellence; optimising our resources	54
Arizona health care cost containment system	Behavioural Health System performance Framework and Dashboard, USA(non-profit-GO)	Not stated	Outcomes; access to services; service delivery; coordination and collaboration	15

Perspectives ranged from the traditional four perspectives devised by Kaplan and Norton to industry-specific perspectives such as clinical quality, patient perception of care, and access to services.^12^ The number of indicators ranged from 11 to 94, with a mean number of 27 indicators per BSC. Strategic goals were varied and represented the broad aims of MHS. These are described in Table 2.

### Themes Identified

From the 17 BSCs identified in this review, 16 BSCs had published the complete tool allowing for indicators to be analysed. A total of 434 indicators were collated and included for thematic analysis. Eleven key themes were identified amongst the indicators, and 16 miscellaneous indicators. Themes consisted of the following:

1. Prevalence: the frequency of mental health conditions of symptoms present in the population. Sixty-two indicators were identified within this theme. Examples include *% hospitalised for psychotic diagnoses* and *Prevalence of self-reported mental illness and substance use for youth.*
2. Accessibility: the rate at which clients can access services due to wait times and other restrictions. From this theme, there were 43 indicators identified. Examples include *Rate at which children and youth were seen by a psychiatrist* and *% of all referrals waiting* ≥ *26 weeks.*
3. Services provided: services and care practices available and provided to clients. Sixty-six indicators were identified within this theme. Examples include *Number of consultations* and *% of Care Plans updated at minimum of once per month*.
4. Clinical outcomes: the clinical outcomes for clients receiving their services. Thirty-nine indicators were identified within this theme. Examples include *% of discharged patients with improved positive symptoms (Schizophrenia)* and *Readmission Rate: % of clients re-admitted to any facility within 30 days of discharge*.
5. Client satisfaction: how satisfied clients were with their experiences with the service. Within this theme, there were 28 indicators identified. Examples include *Client Experience Input Survey % of positive responses to the question 31, I think the services provided here are of high quality* and *% of Service Complaints Received*.
6. Client involvement: client involvement in their care. There were 10 indicators identified within this theme. Examples include *% Hospital or program advisory/steering committees with consumer representation* and *% Discharge plans completed with client involvement.*
7. Staff motivation: factors impacting staff satisfaction such as comparative salaries, training, professional development opportunities and engagement. There were 50 indicators within this theme. Examples include *% of staff who received training* and *Enhance employee motivation and empowerment (new skills, decision-making participants, performance improvement activities).*
8. Staffing levels: this includes staffing ratios, caseloads, and number of staff. Thirty-seven indicators were allocated to this theme. Examples include *Physicians’ full-time equivalent allocation to mental health care for children and youth* and *% staff and staff hours on variable hours.*
9. Governance and compliance: governance related activities and reporting, certification, and compliance with regulations. Allocated to this theme were 25 indicators. Examples include *% case files audited,* and *Medication Incidents per 1000 Patient Days: All Medication Incidents per 1000 patient days reported during the period* and *Serious medication incidents (Moderate, Severe or Death Degrees of Harm as defined by the National System for Incident Reporting) per 1000 patient days reported during the period.*
10. Development: initiates and programs developed to improve functioning of the service or impact. Within this theme, there were 32 indicators identified. Examples include *Development of new roles and support arrangements for sign-off by MHI Board* and *Positive Behaviour Interventions & Supports (PBIS): Status of Implementation.*
11. Costs and revenue: financial balance of the organisation. 44 indicators were allocated to this theme. Examples include *Agency costs* and *Cost-effective service (inpatient costs per day).*

A breakdown of the themes represented by each of the 16 BSCs are represented in Table 4. Some BSCs considered MHS from a range of different angles, such as the BSC of Western Isles Health and Social Care, 2018, which contained indicators from all eleven themes, whilst some BSCs were narrower in focus, comprising a smaller number of themes amongst their indicators, such as the Alaska Mental Health Trust Authority, 2019 with only three themes.

**Table 4:** Analysis of thematic components of current BSC in mental health services

	**Prevalence**	**Accessibility**	**Services provided**	**Clinical Outcomes**	**Client Satisfaction**	**Client involvement**	**Staff motivation**	**Staffing levels**	**Governance compliance**	**Development**	**Cost and revenue**
Coop, 2006	✔	✔	✔	✔	✔		✔	✔			✔
Lin & Durbin, 2008	✔	✔	✔	✔	✔	✔		✔	✔		
Schmidt et al., 2006	✔	✔	✔	✔			✔	✔			✔
Wolferstieg & Dunham, 1998				✔	✔	✔	✔		✔		✔
Yang et al., 2016	✔	✔	✔	✔				✔			
NHS Scotland CAMHS, 2011		✔	✔	✔		✔		✔	✔		✔
Ontario Shores Centre for Mental Health Sciences, 2019	✔	✔	✔	✔	✔			✔	✔		✔
CMHA Toronto, 2011	✔		✔		✔	✔	✔	✔	✔	✔	✔
Western Isles Health and Social Care, 2018	✔	✔	✔	✔	✔	✔	✔	✔	✔	✔	✔
New Path Youth and Family Services, 2018		✔	✔		✔		✔	✔	✔	✔	
Mental Health Ireland; 2017			✔		✔				✔	✔	✔
Alaska Mental Health Trust Authority, 2019	✔	✔									✔
Center for Addiction and Mental Health, The Royal Mental Health Centre, Waypoint, 2019	✔	✔	✔	✔	✔		✔	✔	✔		✔
Griffin Center, 2018		✔	✔		✔		✔		✔	✔	✔
Spectra helpline Canada, 2017		✔	✔		✔		✔	✔	✔	✔	✔
Arizona health care cost containment system	✔	✔	✔	✔	✔					✔	

## Discussion

The authors found that a few mental health focused BSCs had been published in the peer-reviewed literature and that there was an overall failure to describe the impact of utilisation of BSCs on health services. Instead, both peer-reviewed articles and industry reports focused on indicator development or current findings from the service in which the BSC was developed. First-generation BSCs, prioritising the reporting of performance measures across the four domains to meet stakeholder satisfaction without further examination of causal relationships, comprised the majority of BSCs identified. Despite MHS reporting of strategic objectives, indicators, and organisational success within the measures of their BSCs, there was a lack of reporting into perspective hierarchy and causal relationships between measures required for subsequent generations of the scorecard.^16,17^ The impact of the BSC to inform organisational strategy and its overall success as a performance management system cannot be extrapolated from the current lack of rigorous reporting of BSCs incorporation into MHS.

The BSCs identified did allow for thematic analysis of indicators to be conducted and 11 themes were identified to create a comprehensive BSC. These themes together would allow MHS to assess organisational performance from a diverse range of health-related elements. All the BSCs examined contained a range of these themes, with some more ‘balanced’ in their approach than others. The inclusion of particular themes was impacted upon by the strategic objectives of each organisation. For example, organisations that were primarily focused on providing telehealth services, such as Spectra Help Line,^28^ or community services such as Griffin Centre,^29^ did not contain indicators of clinical outcomes. Strategic objectives and operational scope were imperative to identifying relevant indicators.

A focus on balancing judgements of the organisation by leadership, as well as optimising judgements away from traditional financial measures is particularly important in health services in which patient outcomes are significant to strategic goals of the organisation.^18^ BSCs in the health context provide a more comprehensive framework of assessing service delivery and system elements for optimal service provision.^19^ As observed in Table 4, MHS routinely measured performance from a range of health system facets where the impact of direct patient care on patient outcomes are required to meet organisation directed goals. The underlying themes elucidated from the indicators across 16 BSCs analysed in this integrative review highlight how current MHS construct performance measurement frameworks. These themes represent the major service delivery elements and system elements applicable to MHS and provide key areas of performance benchmarking. It can also be inferred that several of the themes would be applicable to other health and aged care services.

### Health Service Specific Indicators

The utilisation of BSC in health industries has primarily been to improve health care quality and to meet national benchmarks on which funding is dependent.^30^ Indicators focusing on the performance of medical treatment and health care typically dominate the internal processes perspective with evidence to indicate that health care specific indicators tend to comprise the majority of the BSC in the broader health care industry.^30,31^ In this integrative review, health service specific indicators represented 221 (50.9%) of total indicators. Prevalence of health conditions or symptoms within the population, accessibility of patient services, health service provision, and clinical outcomes measures may be viewed as core indicators of quality of care and are specific to the health care field.

Client involvement in their care was a theme identified in five of the 16 BSCs reviewed. Client involvement in decision‐making processes is now emphasised in health care systems worldwide as an ethical requirement to empower patients and increase autonomy.^32^ Up until the 1980s mental health clients were only passive recipients in their care until a fundamental shift occurred and clients became involved in their care, actively influencing the services they use.^6^ Client involvement in mental health care can be defined as (1) participation in decision-making; (2) active involvement, rather than merely receiving information; (3) involvement in a diverse range of activities, such as planning, monitoring, and development; (4) acknowledgment of expertise obtained through lived experience; and (5) collaboration with health professionals.^32^ Greater client involvement enhances satisfaction.^33^ BSCs in this study did not adequately cover all facets of client involvement, focusing instead on involvement in decision-making process and planning.

### Adapted General Indicators

BSCs typically include measures of customer satisfaction, learning and growth, internal processes, and financial management. Measures pertaining to these perspectives were found to be inconsistent across included BSCs. This may be attributed to the goals of the respective institutions, as well as limitations in organisational formulated measurement tools.

#### Customer Perspective

There is now increased recognition of the patients’ experiences of mental health services within performance measurement to provide information from the patient/client view about navigating the system, the quality of care they have received, and ultimately their self-reported outcomes.^7^ External customer satisfaction was measured in 12 of 16 of the BSCs, whereas internal customer satisfaction including staff and stakeholder satisfaction was measured in only four. It was observed that 6.4% (*N* = 28) of indicators related to client satisfaction. Client satisfaction in health care is a complex construct.^34^ MHS are multidimensional and client satisfaction is impacted by clients personal attributes as well as health service delivery factors, such as patient-professional interactions, physical environment, and internal management processes.^34,35^ Measures included in the BSC did not reflect client’s preferences even though such information is important for cost-effective decision-making.^35^ Although multiple evidence based tools for measuring health care satisfaction have been reported,^34,35^ these do not appear to have been adapted to organisational measures such as the BSC.

#### Internal Process Perspective

Internal processes within the MHS context reflect the relevant service models of each organisation. High rates of readmission due to poor treatment adherence, substance abuse, and early discharge due to service limitations have promoted a policy shift away from institutionalised care,^36^ and most organisations in this review provided community-based care. Indicators relating to service provision, accessibility, and governance and compliance provide a limited picture of how systems currently operate. Measures of service provision such as discharge plans, service utilisation, and patient length of stay, inform organisations of their capacity to adequately complete care related tasks within an optimal time frame. The ability of organisations to move patients through the system was a significant theme with 13/16 organisations including measures of accessibility. This is important, as effective clinical referral pathways have the capacity to influence patient satisfaction.^37^ MHS typically involve numerous personnel and services which contributes to system complexity that patients have to navigate.^6^ Organisations also considered measures of governance and compliance in 11 of the scorecards. These measures differed due to policy differences relevant to federal, state, and institutional formats and requirements for such reporting in addition to institutional priorities. There may be differences attributable to national goals for mental health care influencing number and type of items included for these performance measures.^38^

#### Financial Perspective

Financial measures are fundamental to the strategic management of health services with financial measures representing essential resources to achieve the patient (customer) and internal process perspectives.^39^ With health services prioritising patient outcomes over profit, the financial perspective more accurately reflects a constraint to achieving key performance indicators rather than a measure of success based on profit. This is particularly pertinent as all MHS utilising the BSC were not-for-profit organisations.^10^ It was observed that financial measures were not prominent throughout the MHS BSCs with only 44 (10.1%) of the total indicators being directly related to costs and revenue. Staffing levels which accounted for 37 (8.5%) of the total indicators also represented financially driven human resources. Indicators, such as *% of staff and staff hours lost to unplanned absence type by locality* and *absence type (sick, LT sick, compassionate/special leave)*, are not specific to the health care industry but are crucial in terms of patient care hours due to the costs of required agency staff to meet staff ratios.

#### Learning and Growth Perspective

To achieve the previous three BSC—customer, internal processes, and financial perspective—organisations must address the gap between current capabilities and systems and the actions required to meet desired targets.^11^ Through investment in staff training, improved information and technology systems, and research and development organisations can enhance value by improving processes, intellectual capital, and performance.^11^ A causal relationship between measures of the learning and growth perspective and measures of the financial perspective have been reported.^40^ Evidence supports a relationship between organisational learning leading to increased efficiency and quality indicating that the more an organisation invests in learning and growth the greater the ability to meet other measures of performance.^40^ For example, embedding of professional development opportunities for employees to enhance their careers and improve leadership skills within long-term strategic plans can enhance employee engagement and improve retentions rates.^41^ The learning and growth perspective for the MHS BSCs reviewed included indicators of development for program and service initiatives and some facets of staff motivation such as staff training and education and participation in research projects. It was observed that only 7 out of 16 (44%) BSCs included staff motivation and 9 out of 16 (56%) BSCs included service and program development suggesting that the learning and growth domain may be underpowered.

## Implications for Behavioural Health

Importantly for mental and behavioural health organisations, this review identifies insufficiencies in performance management and monitoring. Organisations cannot manage what they cannot see. Whilst the BSC methodology has been widely applied in the private for-profit sector, implementation in not-for-profit organisations (NPOs) and public sectors, that encompass most MHS, requires additional emphasis on organisation mission and client outcomes over profit.^10^ Without adequate adaptation of key indicators reflective of the strategic objectives of the health care sector, a BSC in a NPO is a limited measurement tool instead of a fully functioning management system. This may result in negative employee attitudes and poor implementation.^42^ Adaptation of the traditional four perspectives to more accurately reflect health service key performance indicators (KPIs) and aligning indicators with key objectives provides more meaningful information.^43^ These KPIs and the strategic mission of a MHS should be adaptable to external and internal changes in circumstances, such as in the case of COVID-19 pandemic which requires indicators to capture changes relating to telehealth and improved digital health solutions. This review identified 11 key themes that are specific to the health services sector that may inform management and organisational leaders in the construction of a more holistic BSC and highlights current missed opportunities for quality monitoring.

Accessibility and clinical outcomes for most MHS were measured well in most instances; however, current deficits in multidimensional measures of client involvement in care and client satisfaction, and the underestimation of the importance of the learning and growth perspective, highlight areas of future improvement. Mental health NPOs need to recognise and measure intellectual capital and ongoing development opportunities to ascertain a more effective learning and growth perspective.

Additionally, development of an effective BSC relies on staff engagement in development of organisational strategy and priorities. Fulfilling the organisational strategy is the job of every employee, not just for those in upper management positions, and implementation of BSCs in NGOs requires uptake at all organisational levels.^42^ The mission statement, as an overall common identity reflective of both employees and stakeholders and a clear strategy map narrating the desired directions of the community as a whole, has been shown to be significant for the success of implementation of a BSC.^44^ Employees should feel involved in BSC development and supported by executive management.^43^ Additionally, MHS users should be involved in service innovation in order for organisations to provide increased value to the consumer and improve satisfaction.^6^ Future research should address how employees and consumers can shape the development of a BSC system through engagement and continued feedback and how this can improve implementation and organisational success through the meeting of carefully designed objectives.

## Conclusion

Several MHS across the world currently use and publicly release their results on key performance indicators using the BSC approach. Organisations define their key strategic objectives and adapt the BSC to report relevant health-related outcomes. However, there is little research into the implementation of a BSC within the organisation and the impact on organisational strategy and outcomes. Thematic analysis of indicators across the BSCs revealed 11 key themes. These themes are broadly applicable to the MHS and represent a common string of values specific to not-for-profit health services sector. Future development of BSCs may utilise these themes as a base to provide a more balanced measurement tool.

## Declarations

### Disclaimer

A literature review of published journal articles was conducted following the PRIMSA guidelines (Moher, Liberati, Tetzlaff, Altman, & Group, 2009).

## References

[1] Schaefer JD, Caspi A, Belsky DW (2017). Enduring Mental Health: Prevalence and Prediction. Journal of Abnormal Psychology..

[2] Naylor C, Parsonage M, McDaid D, et al. Long-Term Conditions and Mental Health: The Cost of Co-Morbidities. *The King's Fund, London, UK.* ISBN 9781857176339, 2012. Available online at: http://eprints.lse.ac.uk/id/eprint/41873. Accessed 7 Feb 2021.

[3] Australian Institute of Health and Welfare. Mental Health Services in Australia.Web report*. AIHW.* 2019. Available online at: https://www.aihw.gov.au/reports/mental-health-services/mental-health-services-in-australia/report-contents/expenditure-on-mental-health-related-services. Accessed 8 Feb 2021.

[4] Meena K, Thakkar J (2014). Development of Balanced Scorecard for Healthcare Using Interpretive Structural Modeling and Analytic Network Process. Journal of Advances in Management Research..

[5] Hanefeld J, Powell-Jackson T, Balabanova D (2017). Understanding and Measuring Quality of Care: Dealing With Complexity. Bulletin of the World Health Organization..

[6] Sindakis S, Kitsios F (2016). Entrepreneurial Dynamics and Patient Involvement in Service Innovation: Developing a Model to Promote Growth and Sustainability in Mental Health Care. Journal of the Knowledge Economy..

[7] Kilbourne AM, Beck K, Spaeth-Rublee B (2018). Measuring and Improving the Quality of Mental Health Care: a Global Perspective. World Psychiatry..

[8] Brownson RC, Fielding JE, Green LW (2018). Building Capacity for Evidence-Based Public Health: Reconciling the Pulls of Practice and the Push of Research. Annual Review of Public Health..

[9] Saxena S, Lora A, Van Ommeren M (2007). WHO's Assessment Instrument for Mental Health Systems: Collecting Essential Information for Policy and Service Delivery. Psychiatric Services..

[10] Grigoroudis E, Orfanoudaki E, Zopounidis C (2012). Strategic Performance Measurement in a Healthcare Organisation: A multiple Criteria Approach Based on Balanced Scorecard. Omega..

[11] Jelenic D. The Importance of Knowledge Management in Organizations–With Emphasis on the Balanced Scorecard Learning and Growth Perspective. Paper presented at: *Management, Knowledge and Learning, International Conferen*ce, 2011.

[12] Kaplan RS, Norton DP. The Balanced Scorecard: Measures That Drive Performance. *Harvard Business Review.* January-February 1992, pp.71–79. 10119714

[13] Kaplan RS, Norton DP. Using the Balanced Scorecard as a Strategic Management System.*Harvard Business Review*. January-February 1996, pp.1–13.

[14] Voelker KE, Rakich JS, French GR (2001). The Balanced Scorecard in Healthcare Organizations: a Performance Measurement and Strategic Planning Methodology. Hospital Topics..

[15] Perkins M, Grey A, Remmers H (2014). What Do We Really Mean by “Balanced Scorecard”?. International Journal of Productivity and Performance Management..

[16] Bisbe J, Barrubés J (2012). The Balanced Scorecard as a Management Tool For Assessing and Monitoring Strategy Implementation in Health Care Organizations. Revista Española de Cardiología (English Edition)..

[17] Valmohammadi C, Servati A (2011). Performance Measurement System Implementation Using Balanced Scorecard and Statistical Methods. International Journal of Productivity and Performance Management..

[18] Aidemark LG (2001). The Meaning of Balanced Scorecards in the Health Care Organisation. Financial Accountability & Management..

[19] Edward A, Kumar B, Kakar F (2011). Configuring Balanced Scorecards for Measuring Health System Performance: Evidence From 5 Years’ Evaluation in Afghanistan. PLoS Med..

[20] McDonald B. A Review of the Use of the Balanced Scorecard in Healthcare.*Newcastle: BMcD Consulting.* April, 2012. Available online at: https://d1wqtxts1xzle7.cloudfront.net/48009451/Review_of_the_Use_of_the_Balanced_Scorecard_in_Healthcare_BMcD-with-cover-page-v2.pdf?Expires=1644904366&Signature=T3MFHiNVbO2aRgqdchZWmyUP2YpXui6KffQa2BcAS4n4sZ2Ndbc3YTF9MpIivxD3ozFsQ7QTngKPbG3t24gweN~-5MrsFPl4ZPqvT5Kml2E4VkGOH~cY~F-EZQP8SlLodWSkNULhgk~pgYbNXe6hhog0apljgq-jGUxTqbgFXKCQHrv0rQB~f39dKiZNfCaP4G8ZhuRBqqCzHuzi5S6IA5iRJvBYAP6863iUbubK2ajOadWnbyzIpx2-cXALNE8SThg8FyiBi8yvkvENNGnWYXySS9~Hgq0R~dfF7~TcD63DiCyn6rnPIbv7yLAmICNObmOtHBHC4yYQ6v3EjCy9cA__&Key-Pair-Id=APKAJLOHF5GGSLRBV4ZA. Accessed 12 Feb 2021.

[21] Rimar S, Garstka SJ (1999). The “Balanced Scorecard”: Development and Implementation in an Academic Clinical Department. Academic medicine: journal of the Association of American Medical Colleges..

[22] Ten Asbroek A, Arah O, Geelhoed J, et al. Developing a National Performance Indicator Framework for the Dutch Health System. *International Journal for Quality in Health Care.* 2004;16(suppl_1):i65-i71. 10.1093/intqhc/mzh020. 10.1093/intqhc/mzh020 15059989

[23] Biro LA, Moreland ME, Cowgill DE (2003). Achieving Excellence in Veterans Healthcare–a Balanced Scorecard Approach. Journal for healthcare quality: Official Publication of the National Association for Healthcare Quality..

[24] Gurd B, Gao T (2007). Lives in the Balance: An Analysis of the Balanced Scorecard (BSC) in Healthcare Organizations. International Journal of Productivity and Performance Management..

[25] Santiago JM (1999). Use of the Balanced Scorecard to Improve the Quality of Behavioral Health Care. Psychiatric Services..

[26] Moher D, Liberati A, Tetzlaff J (2009). Preferred Reporting Items for Systematic Reviews and Meta-Analyses: The PRISMA Statement. PLoS Med..

[27] Vaismoradi M, Turunen H, Bondas T (2013). Content Analysis and Thematic Analysis: Implications for Conducting a Qualitative Descriptive Study. Nursing & Health Sciences..

[28] SPECTRA. Strategic Plan and Balanced Scorecard.Avaialable at: https://www.spectrahelpline.org/images/PDF/SPECTRAStrategicPlanAndBalancedScorecard2014-2015.pdf. Published 2015. Accessed 3 June 2019.

[29] Griffin Centre. Blanced Scorecard Griffin Centre. Avaialable at: http://www.griffin-centre.org/documents/Balanced%20Scorecard%202017-18_FINAL.pdf. Published 2018. Accessed 3 June 2019.

[30] Kollberg B, Elg M (2011). The Practice of the Balanced Scorecard in Health Care Services. International Journal of Productivity and Performance Management..

[31] Mutale W, Godfrey-Fausset P, Mwanamwenge MT (2013). Measuring Health System Strengthening: Application of the Balanced Scorecard Approach to Rank the Baseline Performance of Three Rural Districts in Zambia. PLoS One..

[32] Tambuyzer E, Pieters G, Van Audenhove C (2014). Patient Involvement in Mental Health Care: One Size Does Not Fit All. Health Expectations..

[33] Dixon LB, Holoshitz Y, Nossel I (2016). Treatment Engagement of Individuals Experiencing Mental Illness: Review and Update. World Psychiatry..

[34] Almeida RSd, Bourliataux-Lajoinie S, Martins M. Satisfaction Measurement Instruments For Healthcare Service Users: A Systematic Review. *Cadernos de Saude Publica.* 2015;31:11–25. 10.1590/0102-311X00027014. 10.1590/0102-311x00027014 25715288

[35] Crow H, Gage H, Hampson S, et al. Measurement of Satisfaction With Health Care: Implications for Practice From a Systematic Review of the Literature. *Health Technology Assessment.* 2002;6(32). Available at: https://uhra.herts.ac.uk/bitstream/handle/2299/1073/102382.pdf. 10.3310/hta6320 12925269

[36] Petersen I, Lund C. Mental Health Service Delivery in South Africa From 2000 to 2010: One Step Forward, One Step Back. *South African Medical Journal.* 2011;101(10):751–757. Available online at: https://www.ajol.info/index.php/samj/article/view/70342. 22272856

[37] Allen J, Annells M, Nunn R (2011). Evaluation of Effectiveness and Satisfaction Outcomes of a Mental Health Screening And Referral Clinical Pathway for Community Nursing Care. Journal of Psychiatric and Mental Health Nursing..

[38] WHO. WHO Country Profiles: Mental Health in Development (WHO proMIND). World Health Organisation. Available online at: https://www.who.int/mental_health/policy/country/countrysummary/en/. Published 2019. Accessed 22 Sept 2019.

[39] Niemiec A. Strategic Map For Hospital Management: Perspectives and Priorities. *Economics & Sociology.* 2016;9(3):63–75. 10.14254/2071- 789X.2016/9–3/6.

[40] Perlman Y (2013). Causal Relationships in the Balanced Scorecard: A Path Analysis Approach. Journal of Management and Strategy..

[41] Bhagra A, Croghan IT, Monson TR (2020). An Innovative, Pilot Program to Enhance Career Development and Staff Engagement for Mid-and Late-Career Physician Staff Within an Academic Institution: The RISE Program. Mayo Clinic Proceedings: Innovations, Quality & Outcomes..

[42] Greiling D (2010). Balanced Scorecard Implementation in German Non-Profit Organisations. International Journal of Productivity and Performance Management..

[43] Seitz V, Harvey C, Ikuma L, et al. A Case Study Identifying Key Performance Indicators in Public Sectors. Paper presented at: IIE Annual Conference. Proceedings. 2014.

[44] Pietrzak M. The Application of a Balanced Scorecard in Higher Education Institutions: A Case Study of Wuls. In: Sinuany-Stern. (eds) Handbook of Operations Research and Management Science in Higher Education. *International Series in Operations Research & Management Science, vol 309*. Springer, Cham ; 2021:419–451. 10.1007/978-3-030-74051-1_14.

